# Zone I Flexor Tendon Injuries

**Published:** 2016-11-12

**Authors:** Evgenios Evgeniou, Harriet Walker

**Affiliations:** ^a^North Bristol NHS Trust, Bristol, United Kingdom; ^b^Plymouth Hospitals NHS Trust, Plymouth, United Kingdom

**Keywords:** hand trauma, flexor tendon injury, closed FDP rupture, zone I, avulsion fracture

## DESCRIPTION

A 25-year-old male student presented with pain and swelling of the right index finger following catching it in another player's T-shirt while playing rugby. On examination, he was unable to flex his distal interphalangeal joint (DIPJ). Plain radiographs revealed an avulsion fracture of the distal phalanx retracted back to the level of proximal interphalangeal joint (PIPJ).

## QUESTIONS

1. **Describe a classification system for zone I flexor tendon injuries.**
2. **What are the options for repair of zone I flexor tendon injuries?**
3. **What are the possible complications of zone I flexor tendon repairs?**
4. **What is the importance of postoperative hand therapy?**

## DISCUSSION

Zone I tendon injuries are relatively common injuries that involve the flexor digitorum profundus (FDP) from its insertion onto the distal phalanx base up to the insertion of the flexor digitorum superficialis onto the base of the middle phanax.^1^ Patients present either at the time of injury or more usually further down the course of injury due to ongoing loss of function. Zone I tendon injuries can be open with division of the flexor tendon^1^ or closed with or without an avulsion fracture. Closed injuries are usually the result of forced extension to a DIPJ that is actively flexing and therefore are frequently seen in injuries sustained through sporting activities such as when a sportsperson grasps the shirt or jersey of an opponent.^2^ For this reason, such injuries are also referred to as a “jersey finger.” The mechanism of open tendon injuries differs from that of closed injuries and is not discussed here. Leddy and Packer^2^ classified closed zone I flexor tendon injuries into 3 categories:

1. No bony fragment, rupture of both vinculae and tendon retraction into palm;
2. Small fragment, with proximal tendon end held by long vinculum at the level of PIPJ; and
3. Large bony fragment caught at A4 pulley and both vinculae intact.
  Smith^3^ suggested an extension of this classification system to include:
4. Intra-articular fracture of the distal phalanx combined with avulsion of the FDP tendon from the avulsed fragment.

Various techniques have been described for the repair of zone I flexor tendon injuries. If there is enough distal tendon, a primary tendon repair can be performed using conventional techniques such as modified Kessler technique.^1^ If there is not enough distal tendon for a primary tendon repair or in cases of FDP avulsion injuries, the FDP tendon needs to be reattached to the distal phalanx. Various techniques for tendon reattachment have been described in the literature such as the use of a bone anchor, button pullout techniques,^4^ or intraosseous fixation techniques.^5^

General complications following zone I flexor tendon repairs include infection, scar, stiffness, and reduced range of movement (ROM) due to joint contractures or adhesions, damage to other structures such as nerves and vessels, rupture of the repair, and complex regional pain syndrome. Tendon reattachment techniques using bone anchors have the added risks of foreign body reactions, extrusion of the foreign material, or detachment of the bone anchor.^5^ Button pullout techniques, which are now rarely being used, can lead to infection because the suture material is exposed and can cause damage to the germinal matrix with subsequent effects on nail growth.^4^

Hand therapy following flexor tendon repairs is a balance between allowing the tendon to heal and preventing the formation of adhesions that can compromise postoperative ROM. Hand rehabilitation regimens can be categorized into delayed mobilization, which are now rarely being used because of the high incidence of adhesions, and early passive or early active mobilization. The Kleinert protocol comprises active extension and passive flexion exercises using the resistance from elastic bands.^6^ Controlled active motion protocols use supervised early active flexion and extension exercises^7^ and have been shown to produce better outcomes than early passive mobilization regimens.^8^

Zone I flexor tendon injuries are relatively common and their management can be challenging, especially if these involve reattaching the tendon onto the distal phalanx. Postoperative physiotherapy with early mobilization regimens is crucial to achieve the best possible functional outcome.

## Figures and Tables

**Figure 1:**
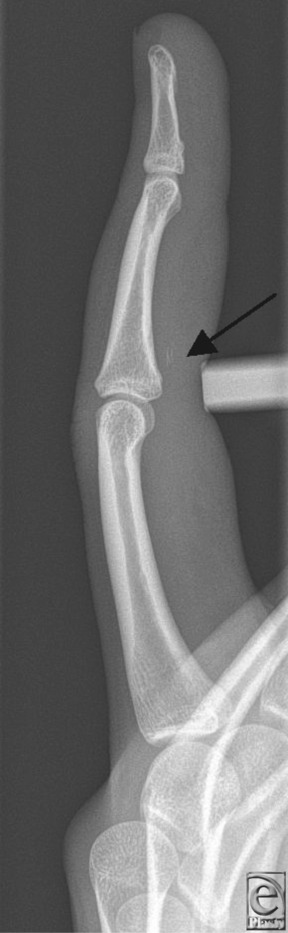
Image demonstrating a hyperextended distal interphalangeal joint and a small avulsion fragment (marked) held at the proximal interphalangeal joint.

**Figure 2:**
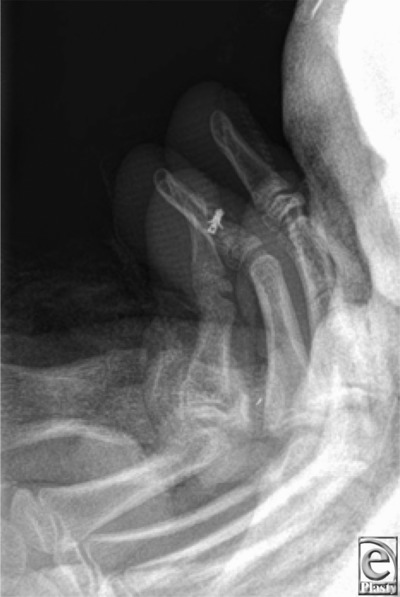
Image post fixation of the avulsed flexor digitorum profundus tendon with a Mitek bone anchor.
